# Intrinsic dynamics study identifies two amino acids of TIMP-1 critical for its LRP-1-mediated endocytosis in neurons

**DOI:** 10.1038/s41598-017-05039-z

**Published:** 2017-07-14

**Authors:** Laurie Verzeaux, Nicolas Belloy, Jessica Thevenard-Devy, Jérôme Devy, Géraldine Ferracci, Laurent Martiny, Stéphane Dedieu, Manuel Dauchez, Hervé Emonard, Nicolas Etique, Emmanuelle Devarenne-Charpentier

**Affiliations:** 1 CNRS UMR 7369: Matrice Extracellulaire et Dynamique Cellulaire (MEDyC), UFR Sciences Exactes et Naturelles, Université de Reims Champagne-Ardenne (URCA), Laboratoire SiRMa - Campus Moulin de la Housse, BP 1039, 51687 Reims cedex, France; 2 Plate-forme de Modélisation Moléculaire Multi-échelle (P3M), Université de Reims Champagne-Ardenne, Reims, France; 3 Aix-Marseille Université, CNRS, Centre de Recherche en Neurobiologie et Neurophysiologie de Marseille (CRN2M), UMR 7286, Plate-forme de Recherche en Neurosciences (PFRN), Marseille, France

## Abstract

The tissue inhibitor of metalloproteinases-1 (TIMP-1) exerts inhibitory activity against matrix metalloproteinases and cytokine-like effects. We previously showed that TIMP-1 reduces neurite outgrowth in mouse cortical neurons and that this cytokine-like effect depends on TIMP-1 endocytosis mediated by the low-density lipoprotein receptor-related protein-1 (LRP-1). To gain insight into the interaction between TIMP-1 and LRP-1, we considered conformational changes that occur when a ligand binds to its receptor. TIMP-1 conformational changes have been studied using biomolecular simulations, and our results provide evidence for a hinge region that is critical for the protein movement between the N- and C-terminal TIMP-1 domains. *In silico* mutants have been proposed on residues F12 and K47, which are located in the hinge region. Biological analyses of these mutants show that F12A or K47A mutation does not alter MMP inhibitory activity but impairs the effect of TIMP-1 on neurite outgrowth. Interestingly, these mutants bind to LRP-1 but are not endocytosed. We conclude that the intrinsic dynamics of TIMP-1 are not involved in its binding to LRP-1 but rather in the initiation of endocytosis and associated biological effects.

## Introduction

The tissue inhibitor of metalloproteinase-1 (TIMP-1) is a natural inhibitor of matrix metalloproteinases (MMP) and several a disintegrin and metalloproteinases (ADAM)^1^. Due to its inhibitory functions, TIMP-1 is largely involved in the control of extracellular matrix remodelling in both physiological and pathological conditions^2^. Moreover, TIMP-1 has been widely depicted as a cytokine-like effector that triggers various cellular responses independently of its MMP inhibitory activity^3–5^. For instance, we recently showed that TIMP-1 decreased neurite outgrowth in cortical neurons and that this effect was mainly dependent on its endocytosis mediated by the low-density lipoprotein receptor-related protein-1 (LRP-1)^6^. LRP-1 exhibits important endocytic and signalling functions that regulate the behaviour of many cell types^7^. In neurons, LRP-1 is abundantly expressed and mediates the endocytosis of various extracellular ligands including TIMP-1^8^. High levels of TIMP-1 are secreted by hyperactive astrocytes, and TIMP-1 expression is highly correlated with various neurological diseases^9, 10^. Characterising TIMP-1/LRP-1 interaction could thus be of physiological relevance in the treatment of certain neurodegenerative disorders.

Molecular docking is the traditional method for predicting how a ligand binds a receptor^11, 12^. Nevertheless, this method requires the determination of both partner structures, which is usually done by X-ray diffraction or nuclear magnetic resonance (NMR) spectroscopy. The well-characterised structure of TIMP-1 consists of six disulfide-bonded loops forming two structurally distinct domains, and the three-dimensional TIMP-1 structure has a wedge-shaped appearance^13, 14^. The N-terminal domain (N-TIMP-1), which is composed of 126 amino acids, carries the inhibitory activity against MMPs by forming a non-covalent 1:1 stoichiometric complex with the proteinase^15^. The C-terminal domain (C-TIMP-1), which is composed of 58 amino acids, is structurally less characterised, but it has been shown to interact with the proMMP-9 hemopexin domain^14^.

LRP-1 is a large receptor composed of a long extracellular α-chain non-covalently associated to a short transmembrane β-chain^7^. The α-chain contains four ligand-binding domains composed of cysteine-rich complement-type repeats, and domains II and IV are the major binding regions, interacting with more than forty ligands^7, 16^. Unfortunately, the high molecular mass of LRP-1 and the presence of a predicted unordered region are major obstacles to elucidate the entire LRP-1 structure. Consequently, the use of molecular docking tools is not appropriate for studying the TIMP-1/LRP-1 interaction.

Protein conformational changes defined by protein flexibility and dynamics play a crucial role in ligand/receptor interaction^17–19^. We have thus hypothesised that the alteration of these properties could modify the TIMP-1/LRP-1 interaction and associated cellular effects. Protein dynamics can be evaluated *in silico* by normal mode analysis (NMA)^20, 21^ and/or principal component analysis (PCA) of molecular dynamics (MD) simulations^22^. For instance, combining these approaches helped us to obtain reliable results consistent with the experimental data in the case of CD47/TSP-1 interaction by identifying large amplitude motions of the TSP-1 C-terminal domain^23, 24^. We have thus combined NMA and MD simulations with biological experiments to characterise the TIMP-1/LRP-1 interaction. NMA performed on the structure energy of TIMP-1 showed movement between the N- and C-terminal domains of TIMP-1 and indicated regions with high deformation energy and low carbon alpha atomic fluctuation. NMA and MD clearly pointed out that these regions are located in a hinge region that could be essential for protein movement. Interestingly, a single mutation of residue F12 or K47 (numbering of residues in the mature secreted protein) located in this region inhibits TIMP-1 cytokine-like activity in neurons but surprisingly does not alter TIMP-1 binding to LRP-1 domains II and IV. The data obtained using *in silico* simulations and biological experiments highlight the relevance of protein dynamics in the TIMP-1/LRP-1 interaction and associated biological effects.

## Results and Discussion

### Determination of TIMP-1 intrinsic dynamics using molecular modelling

**Figure 1:**
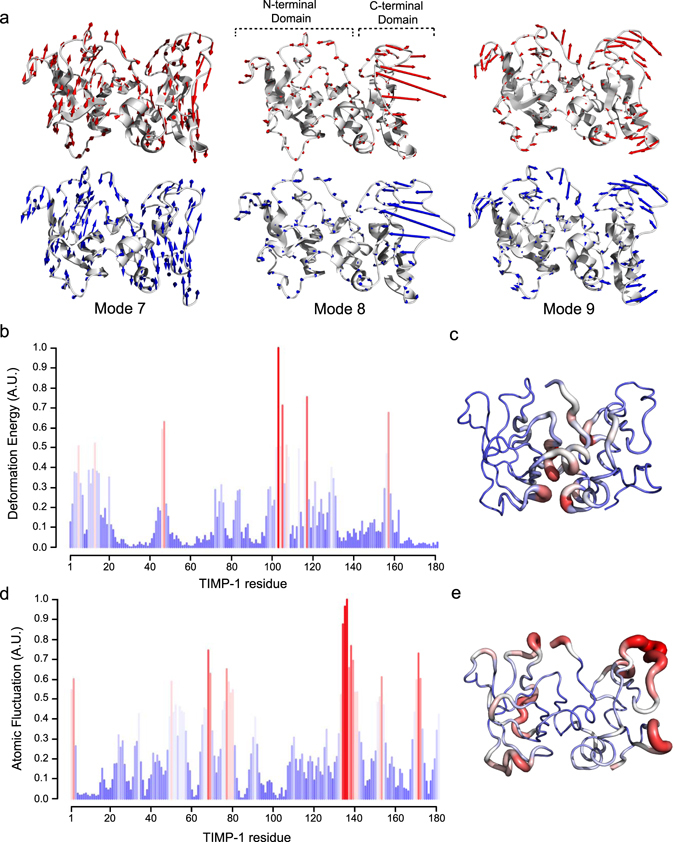
Normal mode analysis of TIMP-1 structure. (**a**) Intrinsic dynamic of TIMP-1 along modes 7, 8 and 9. Open structures of TIMP-1 are in the upper part of the panel and closed structures are in the lower part. Arrows (blue and red) indicate the direction and the deviation (length of arrows) of each residue returning to the 3D reference structure of TIMP-1. (**b**) Deformation energy of each TIMP-1 residue (numbering of the mature secreted protein) along mode 7. The lowest deformation energies are coloured blue and the highest are red. (**c**) Visualisation of the TIMP-1 3D-structure of each amino acid deformation energy. Residues with the lowest deformation energy are thin and coloured blue and those with the highest atomic fluctuation are thick and coloured red. (**d**) Cα atomic fluctuation of each TIMP-1 residue (numbering of the mature secreted protein) along mode 7. The lowest atomic fluctuations are coloured blue and the highest are red. (**e**) Visualisation of the TIMP-1 3D-structure of each amino acid atomic fluctuation. Residues with the lowest atomic fluctuations are thin and coloured blue and those with the highest atomic fluctuations are thick and coloured red.

TIMP-1 has been described as a protein whose function could be controlled by its intrinsic dynamics^25^. Protein dynamics, which represent intrinsic subregional motions, could also be a factor in ligand binding to its receptor^17, 18^. We have thus hypothesised that alteration of these protein dynamics could modify the TIMP-1/LRP-1 interaction. We first studied TIMP-1 intrinsic molecular motion using NMA. The energy of the TIMP-1 structure (PDB 1UEA) after the addition of a hydrogen atom was minimised by successively combining the Steepest Descent and Adopted Basis Newton-Raphson (ABNR) methods. The NMA of this structure generated a set of conformations. We have excluded the first six modes characterised by a null frequency corresponding to rigid-body rotation and translation movements^26^. Of the next following modes, modes 7 to 9 were characterised by the lowest frequencies corresponding to large movements, which are the most functionally relevant for proteins^26^ (Fig. 1a). Moreover, large region displacements are associated with a high degree of collectivity^19^. Taking into account the intermediate structures of TIMP-1 along the three modes, the flexibility profile along mode 7 exhibited the highest degree of collectivity (numerous arrows with similar lengths). This mode, which is presented in the supplementary Movie 1, showed large interdomain displacements comparable to a “wirecutter” movement between the N- and C-terminal domains.

**Figure 2:**
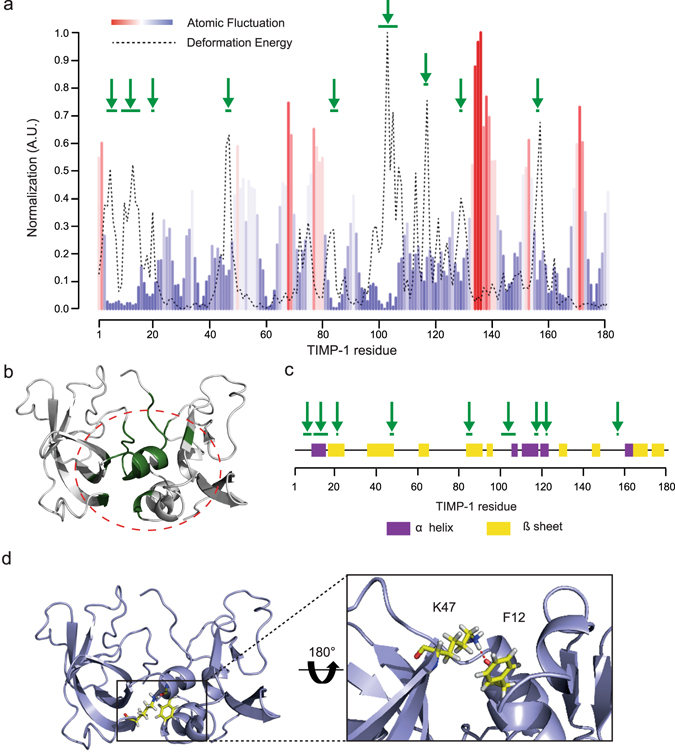
Identification of residues involved in TIMP-1 interdomain movements. (**a**) Superposition of the deformation energy (dotted line) and atomic fluctuation (coloured bars) data from TIMP-1 residues (numbering of the mature secreted protein) along mode 7. The green arrows point out residues or sets of residues with both high deformation energy and low Cα atomic fluctuation. (**b**) Localisation in the TIMP-1 3D structure of the residues identified in (**a**) with both high deformation energy and low Cα atomic fluctuation. These residues are in an area surrounded by a dotted line. (**c**) Localisation in the TIMP-1 secondary structure of the residues identified in (**a**) with both high deformation energy and low Cα atomic fluctuation (green arrows). α helices are defined by the purple area and β sheets by the yellow area. (**d**) Left: localisation in the TIMP-1 3D structure of F12 and K47 residues. Right: hydrogen bond formed between the carbonyl oxygen of the F12 backbone and the K47 side chain.

**Figure 3:**
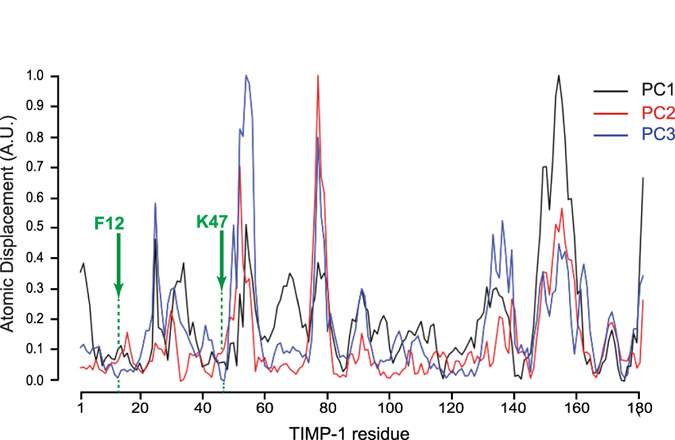
Principal component analysis derived from molecular dynamics simulation of TIMP-1. For each PC, the contribution of each residue to the overall atomic displacement was determined. The green arrows point out residues F12 and K47.

Along mode 7, we determined the associated deformation energy and alpha carbon (Cα) atomic fluctuation to identify the TIMP-1 regions that govern this movement. Deformation energy analysis was used to measure the amount of local flexibility in the protein structure (*i*.*e*. atomic motion relative to neighbouring atoms)^27, 28^. High deformation indicates flexible regions, and our data showed that residues with the highest deformation energy (red bar) were present throughout the primary structure (Fig. 1b). In the TIMP-1 three-dimensional (3D) structure, these residues are located between the N- and C-terminal domains (Fig. 1c). Cα atomic fluctuations provide amplitudes of the absolute atomic motion^28^, and our data (Fig. 1d) showed that residues with high fluctuation (red bar) were dispersed in the TIMP-1 primary structure but located outside the 3D structure (Fig. 1d and e). Interestingly, regions with the highest deformation energy seemed to be localised in the regions exhibiting the lowest atomic fluctuation (Fig. 1c and e). To confirm this observation, the deformation energy and atomic fluctuation data have been normalised and then superposed (Fig. 2a). The graph shows nine regions (green arrow) in which the residues exhibited both high deformation energy and low Cα atomic fluctuation. Interestingly, these regions were grouped in an area of the TIMP-1 3D structure that could be linked to a hinge essential for the interdomain displacements (Fig. 2b). Among the residues present in the nine regions mentioned above, we sought to identify those with mutations that could disturb the movement. Here, we excluded the amino acids present in secondary structures in order to prevent protein misfolding (Fig. 2c). Residues K47 and F12 of the mature secreted protein seemed to be of particular interest because the K47 side chain forms a hydrogen bond with the carbonyl oxygen of the F12 backbone (distance < 3.2 Å). Moreover, this bond was maintained in all intermediate TIMP-1 structures along mode 7 (Fig. 2d), suggesting that these two amino acids could contribute to chain flexibility and N- and C-terminal interdomain displacement. To support this finding, a 100 ns MD simulation was carried out and subjected to PCA to identify collective motions after fitting the trajectory on the first reference frame (Fig. 3). Each PCA showed that F12 and K47 are located outside regions displaying large atomic displacements. Moreover, F12 and K47 along the third PC made the lowest contribution to the total atomic displacement. This additional analysis confirmed that F12 and K47 could be of interest for TIMP-1 movement. We hypothesised that disrupting the interaction between F12 and K47 could affect the interdomain movements of TIMP-1 and its subsequent biological activity.

### Generation of TIMP-1 mutants

**Figure 4:**
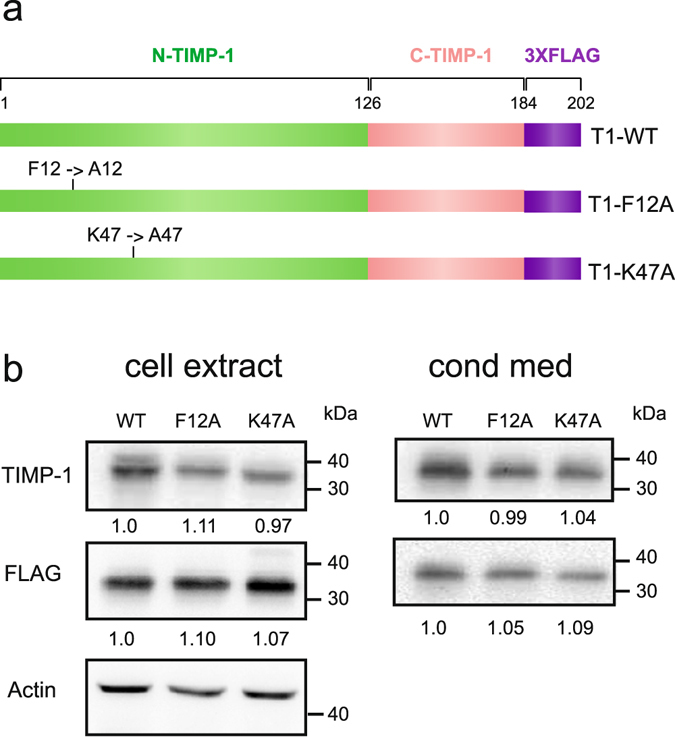
Production of TIMP-1 mutants. (**a**) Schematic representation of T1-WT, T1-F12A and T1-K47A proteins. (**b**) A representative western blot with the indicated antibodies of cell extracts and conditioned medium (cond. med) of stably transfected CHO cells with p3X-FLAG-CMV-14 expressing T1-WT, T1-F12A or T1-K47A. The relative protein level based on three independent experiments is indicated under each band. The gels were run under the same experimental conditions and were shown as cropped gels/blots (Cropped gels/blots are shown in Supplementary Figure S1).

To disrupt the interaction between F12 and K47, these residues were mutated into alanine. The choice of alanine was based on its low steric hindrance and/or its apolar character. This mutation should prevent hydrogen bond formation between K47 and F12 and theoretically disturb the movement described by mode 7. The two mutated TIMP-1 sequences have been inserted into the p3X-FLAG-CMV-14 vector to produce proteins with a C-terminal 3X-FLAG tag, facilitating their purification (Fig. 4a). Each mutant (T1-F12A and T1-K47A) and a wild-type TIMP-1 (T1-WT) were produced in Chinese hamster ovary (CHO) cells to be correctly folded and glycosylated. Western blot analysis using antibodies directed against TIMP-1 or 3X-FLAG showed that T1-WT and TIMP-1 mutants exhibited migration profiles that correspond to the expected 37 kDa molecular mass of the 3X-FLAG glycosylated proteins (Fig. 4b, left panel). Non-transfected CHO cells did not express endogenous TIMP-1 (data not shown) while CHO transfected cells expressed and secreted recombinant T1-WT, T1-K47A or T1-F12A with homogenous expression levels (Fig. 4b, right panel). To ensure proper folding of the recombinant proteins, we tested their ability to inhibit MMP-1, -2, -3 and -9. Indeed, it has been demonstrated that correct folding of TIMP-1 is necessary for its MMP inhibitory activity and that a single point mutation in the TIMP-1 sequence may alter this activity^14, 25, 29^. Our data showed that the Ki values exhibited by mutants were similar to those of T1-WT (Table 1).

**Table 1:** Inhibition constants (Ki) of TIMP-1 mutants against MMP-1, MMP-2, MMP-3 and MMP-9.

	Ki (nM)			
	MMP-1	MMP-2	MMP-3	MMP-9
T1-WT	4.03 ± 1.57	0.09 ± 0.04	1.18 ± 0.47	4.25 ± 0.28
T1-F12A	3.82 ± 1.33	0.06 ± 0.02	0.58 ± 0.60	4.76 ± 0.57
T1-K47A	3.38 ± 2.92	0.03 ± 0.01	0.55 ± 0.19	4.40 ± 0.78

Mean values ± standard error are presented.

Data are based on three independent experiments.

These data indicate that individual single point mutations (K47A and F12A) do not modify the inhibitory activity of TIMP-1 towards these MMPs and confirms that the folding of the two TIMP-1 mutants was correct.

### Effect of the TIMP-1 mutants on neurite outgrowth

**Figure 5:**
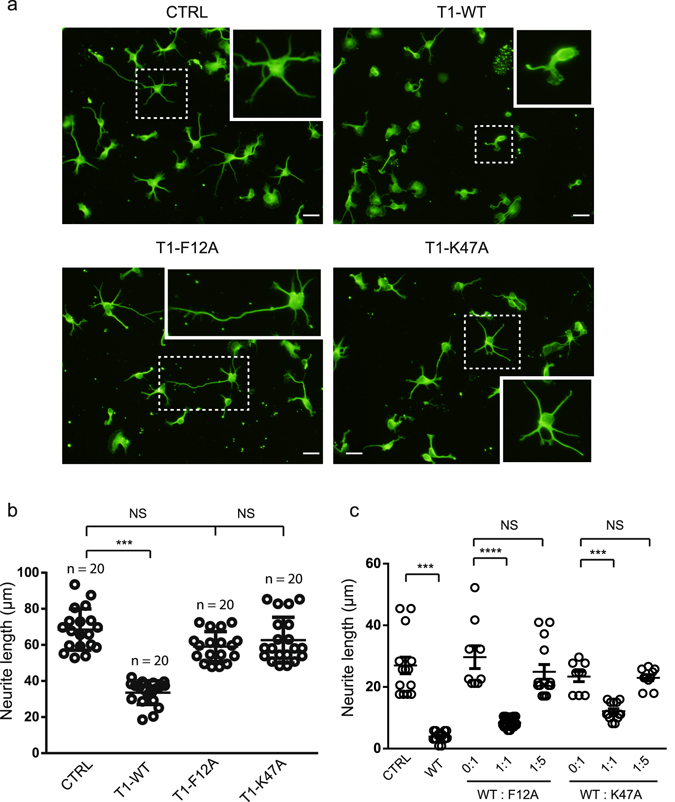
Effect of TIMP-1 mutants on neurite outgrowth. (**a**) Immunofluorescence of cortical neurons from mouse embryos cultured for 24 h on poly-L-lysine-coated coverslips and then treated for 30 min with PBS (CTRL) or 5 nM T1-WT, T1-F12A and T1K47A. Cells were labelled with anti-βIII-tubulin monoclonal antibody and observed by fluorescent microscopy. The images are representative of more than three distinct experiments. Scale bar: 10 µm. (**b**) Quantification of neurite mean length per cell performed using the ImageJ plugin NeuronJ. For each group, at least 20 randomly selected fields were analysed in three independent experiments (n = 20). The data are representative of at least three independent experiments with at least five mice embryos. Error bars indicate mean ± SD, *** indicates P-value < 0.00 and NS indicates a value that is not statistically significant. (**c**) Neurite mean length of cortical neurons from mouse embryos cultured for 24 h on poly-L-lysine-coated coverslips and then treated for 30 min with PBS (CTRL) or T1-WT or co-treated with different T1-WT/T1-F12A or T1-WT/T1-K47A ratios (0:1, 1:1 and 1:5). Cells were labelled with anti-βIII-tubulin monoclonal antibody, observed by fluorescent microscopy and analysed with the ImageJ plugin NeuronJ Quantification. For each group, at least 10 randomly selected fields were analysed (n = 10). The data are representative of at least three independent experiments with at least five mice embryos. Error bars indicate mean ± SEM, *** indicates P-value <0.001 and NS indicates a value that is not statistically significant.

We then evaluated the effect of F12A and K47A mutations on the cytokine-like TIMP-1 activity in mouse cortical neurons. Indeed, we and others have reported that TIMP-1 directly bound to LRP-1 at the plasma membrane of mouse cortical neurons, leading to cell morphology changes^6, 30^. We first evaluated the effects of TIMP-1 mutants on neuron morphology in similar experimental conditions, as previously described^6^. Cortical neurons treated or not treated with T1-WT, T1-F12A or T1-K47A were labelled with anti-βIII tubulin antibodies to visualise the microtubule cytoskeleton and to measure neurite outgrowth (Fig. 5a). As expected, T1-WT significantly reduced the neurite length after 30 min treatment (Fig. 5a and b). In contrast, T1-F12A and T1-K47A exerted no effect on cortical neuron morphology (Fig. 5a and b). To explain this result, it was hypothesised that the TIMP-1 mutant binding to LRP-1 was impaired. To test this, we evaluated the ability of T1-F12A and T1-K47A to interact with LRP-1 domains II and IV by surface plasmon resonance (SPR). Surprisingly, this analysis demonstrated a direct interaction between T1-F12A or T1-K47A and LRP-1 domains II (DII) and IV (DIV), with a nanomolar range of affinity similar to that of T1-WT (Table 2). T1-F12A and T1-K47A were associated with DII about 10 times faster than with DIV, while they dissociated with DII slower compared to DIV (Table 2). The measured association (*k*_*on*_) and dissociation (*k*_*off*_) rates were similar to those previously obtained for wild-type TIMP-1^6^. Our SPR data demonstrated that the lack of neurite outgrowth inhibition could not be explained by a difference of affinity towards LRP-1 between the mutants and T1-WT.

**Table 2:** Surface plasmon resonance data for direct binding of TIMP-1 mutants to immobilised LRP-1 domains II (DII) and IV (DIV).

	*k*_*on*_ (0.106 M^−1^s^−1^)	*k*_*off*_ (0.10^−2^ s^−1^)	*K*_*D*_ (nM)
**DII**			
T1-WT	1.28 ± 0.35	1.36 ± 0.20	10.8 ± 2.1
T1-F12A	1.07 ± 0.25	1.92 ± 0.18	18.5 ± 4.6
T1-K47A	0.70 ± 0.44	8.30 ± 0.78	10.4 ± 4.5
**DIV**			
T1-WT	0.148 ± 0.001	0.56 ± 0.020	37.4 ± 1.7
T1-F12A	0.143 ± 0.006	0.55 ± 0.039	38.7 ± 3.8
T1-K47A	0.427 ± 0.300	1.14 ± 0.830	26.5 ± 0.9

Data are based on three measurements using five different concentrations for each measurement.

The equilibrium constants of dissociation (*K*_*D*_) were calculated from the association (*k*_*on*_) and dissociation (*k*_*off*_) rate constants.

Mean values ± standard error are presented.

To support these unexpected results, we performed a competition assay by co-treating cortical neurons with different T1-WT/T1-F12A or T1-WT/T1-K47A ratios (Fig. 5c). The equimolar ratio of T1-WT/T1-F12A or T1-WT/T1-K47A reduced the effect of T1-WT on neurite length by about twofold. Moreover, a five-fold excess of mutants compared to TIMP-1 completely abrogated the TIMP-1-WT effect. These data indicated that wild-type TIMP-1 and its mutants interacted with identical, or at least spatially similar, amino acids of the LRP-1 α-chain. Intriguingly, although both TIMP-1 mutants bound to LRP-1, F12A and K47A mutations prevented the biological effects associated with the TIMP-1/LRP-1 interaction.

### Study of LRP-1-mediated endocytosis of the TIMP-1 mutants

**Figure 6:**
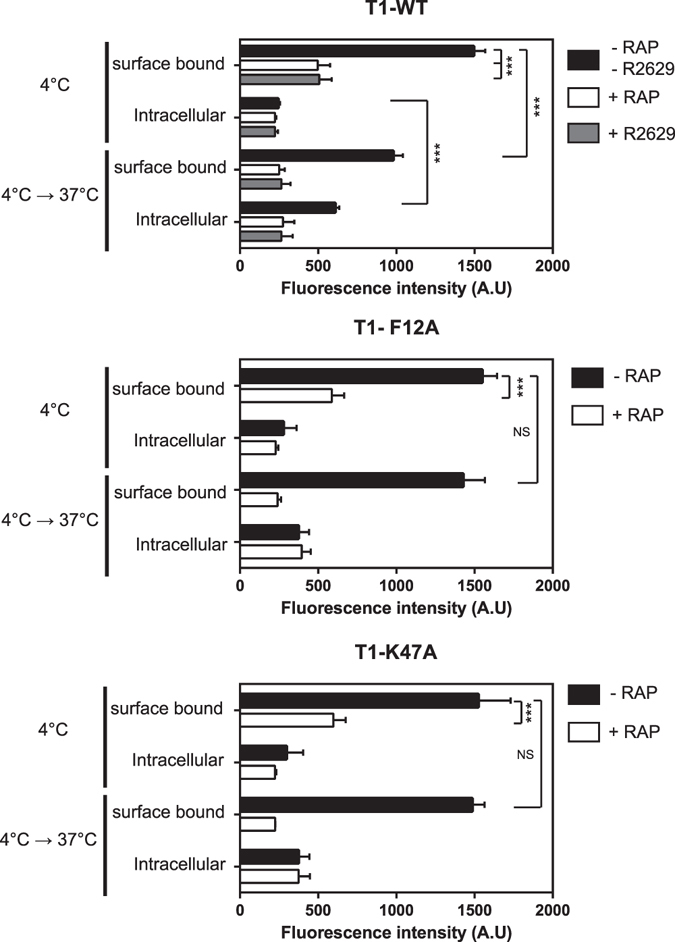
Biochemical analysis of LRP-1-mediated endocytosis of wild-type TIMP-1 and mutants. Cortical neurons from mouse embryos cultured for 48 h on poly-L-lysine-coated coverslips were incubated at 4 °C for 1 hour with 5 nM fluoT1-WT, fluoT1-F12A or fluoT1K47A in the presence or absence of 500 nM RAP or 30 µg/mL of blocking LRP-1 polyclonal antibodies (R2629). After careful washing, part of the cells was used to quantify the surface bound and intracellular signal at 4 °C. The other part was then incubated at 37 °C for an additional 10 min to quantify the surface bound and intracellular signal. Fluorescence intensity was quantified by spectrophotometry and expressed as arbitrary units (A.U.). Values below 300 A.U. are considered to be nonspecific. Error bars indicate mean ± SD, *** indicates significantly different with a P-value < 0.001 and NS indicates a value that is not statistically significant.

LRP-1-mediated endocytosis and signalling are associated events^31^. Since TIMP-1 mutants bind to LRP-1 without triggering a biological effect in neurons, we investigated whether these mutants were internalised by LRP-1 utilising two experimental methods. First, we used eFluor®-conjugated T1-WT (fluoT1-WT), T1-F12A (fluoT1-F12A) and T1-K47A (fluoT1-K47A) according to a previously reported experimental protocol (6) to discriminate cell surface bound from internalised proteins (Fig. 6). At 4 °C, when endocytosis was blocked, we observed that the fluoT1-WT bound to the cell surface of the neurons. This binding was greatly inhibited by RAP, a potent antagonist of ligand binding to LRP receptors^32, 33^ or R2629, a previously validated LRP-1 blocking antibody^34^. The fluorescence at the cell surface was quite similar at about 1500 A.U. for the T1-WT and mutants and confirmed the SPR data (Table 2). RAP similarly impeded the binding of the two eFluor®-conjugated TIMP-1 mutants to the cell surface of neurons. The very low fluorescence intensities measured at 4 °C in the intracellular compartment are considered to be nonspecific. At 37 °C, when endocytosis was initiated, about 30% of the fluoT1-WT bound to the surface of neurons was internalised. Interestingly, the fluorescence intensity at the cell surface remained equal at 4 °C and 37 °C for the two mutants. This result suggests that these mutants were not endocytosed by LRP-1.

**Figure 7:**
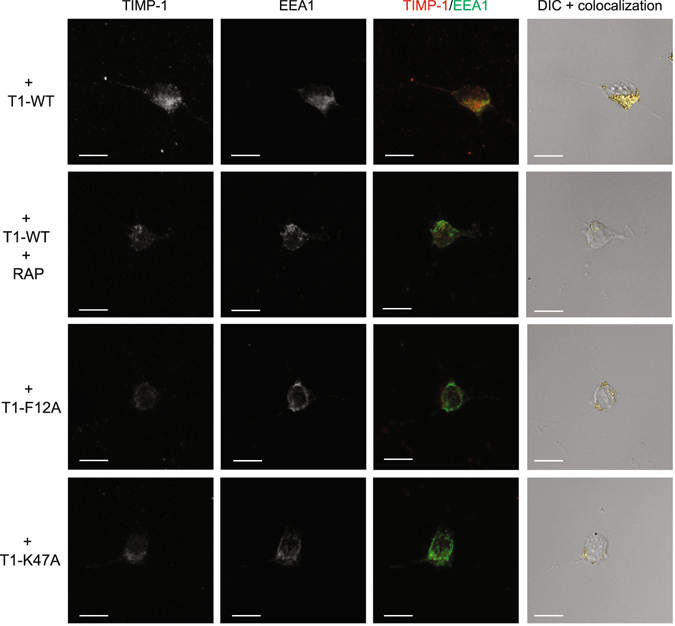
Confocal analysis of LRP-1-mediated endocytosis. Cortical neurons from mouse embryos cultured for 24 h on poly-L-lysine-coated coverslips were incubated at 4 °C for 30 min with 5 nM T1-WT, T1-F12A or T1K47A in the presence or absence of 500 nM RAP and then incubated at 37 °C for 10 min. Cells were stained with Alexa Fluor 488 for TIMP-1 and Alexa Fluor 568 for EEA1 and analysed by confocal microscopy. Images were treated with AMIRA software. Maximal Intensity Projection (MIP) in greyscale of TIMP-1 labelling (first panel) and EEA1 labelling (second panel), TIMP-1/EEA1 merge (third panel, TIMP-1 in red and EEA1 in green), differential interference contrast (DIC) and isosurface representation of TIMP-1/EEA1 colocalisation (fourth panel) are shown. Images are representative of more than three distinct experiments. Scale bar: 10 µm.

**Figure 8:**
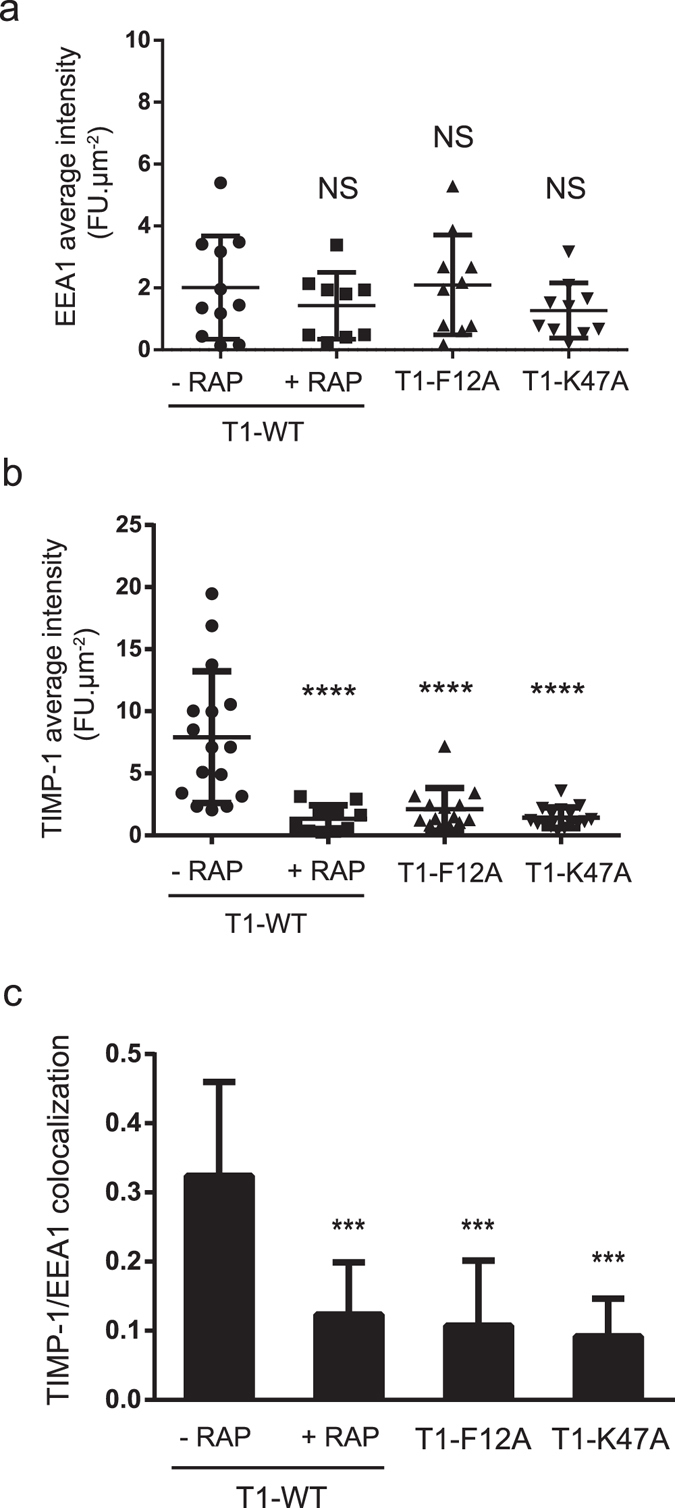
Quantification of TIMP-1 and EEA1 labelling. (**a**) Quantification of average intensity of TIMP-1 labelling per µm^2^ for each cell (n > 10) using ImageJ software. The data are representative of at least three independent experiments with at least five mice embryos. Error bars indicate mean ± SD and **** indicates significantly different from T1-WT without RAP and with a P-value < 0.0001. (**b**) Quantification of average intensity of EEA1 labelling per µm^2^ for each cell (n > 10) using ImageJ software. Error bars indicate mean ± SD and NS indicates a value that is not statistically significant. (**c**) Quantification of TIMP-1/EEA1 colocalisation (n > 10) calculated using AMIRA software. Error bars indicate mean ± SD and *** indicates significantly different from T1-WT without RAP with a P-value < 0.001.

To support these data, we then examined the T1-F12A and T1-K47A internalisation by confocal microscopy imaging (Fig. 7). For this purpose, cortical neurons were incubated with T1-WT, T1-F12A or T1-K47A for 1 h at 4 °C, and then transferred to 37 °C for 10 min to allow endocytosis. Since LRP-1 ligands are internalised in the endosomal compartments^7, 35^, cortical neurons were stained with antibodies directed to TIMP-1 and EEA1, an early endosome membrane marker^36^ (Fig. 7). Confocal imaging analysis revealed that EEA1 labelling was statistically constant in all treated cells (Fig. 8a). T1-WT accumulated inside the cells (Fig. 8b) and co-located with EEA1 (Fig. 8c), demonstrating that wild-type TIMP-1 was actively endocytosed in cortical neurons. RAP inhibited this effect, confirming that TIMP-1 endocytosis was mediated by LRP-1. Interestingly, TIMP-1 labelling was significantly lessened in mutant-treated cells as compared to T1-WT-treated cells (Fig. 8b). Moreover, TIMP-1/EEA1 colocalisation was also decreased in T1-F12A- or T1-K47A-treated cells (Fig. 8c).

Since LRP-1 has been described as a fast endocytic receptor^37^, a rapid cycle of TIMP-1 uptake, trafficking to the cell surface and exocytosis could explain our data. Since our SPR data show that the association and dissociation rates were similar between the T1-WT and mutants (Table 2), this hypothesis is barely conceivable. Another explanation could be a delay of TIMP-1 mutant internalisation. To test this, the endocytosis assay was extended up to one hour, and in this condition, the TIMP-1 mutants were not endocytosed (data not shown). Together, these data highlighted that F12A or K47A mutation prevents TIMP-1 endocytosis in primary cortical neurons.

Several studies have revealed that phosphorylation of the tyrosine residue in the NPXY motifs present in the β-chain intracellular tail represents a molecular mechanism for switching LRP-1 function from that of an endocytic receptor to that of a signalling receptor by modulating the class and type of adaptor proteins that will associate with these motifs^38, 39^. The mechanism leading to the phosphorylation of YXXL and NPXY motifs following ligand binding is still poorly understood. Therefore, it is difficult to explain why TIMP-1 mutants that bind to LRP-1 are not internalised and do not trigger cellular effects in neurons.

Our biological results are in accordance with those of several studies showing that proteins may undergo conformational changes following their binding^40–42^. They confirm our hypothesis that disrupting the interaction between F12 and K47 affects TIMP-1 dynamics and its subsequent biological activity. It would be interesting to use other approaches to confirm that the intrinsic dynamics of F12A and K47A mutants are disturbed. For example, NMR spectroscopy is a powerful technique for measuring protein dynamics. Nevertheless, this technique is restricted by some practical limitations (time cost, quantity of material, protein size). Recent advancements in computational methods allow biomolecular simulation to be considered as an alternative to experimental structural procedures. Consequently, *in silico* approaches remain a reliable way to describe and predict protein movements in association with biological experiments.

The characterisation of the TIMP-1/LRP-1 interaction could be physiologically relevant in some neurodegenerative disorders. Molecular docking is the classical method used to predict how a ligand binds a receptor. However, this method necessitates the structural determination of both partners, and the high molecular weight of LRP-1 excludes the use of this method. In this study, we combined NMA and MD simulations with biological experiments to gain insight into the TIMP-1/LRP-1 interaction. We observed large displacement between the N- and C-terminal TIMP-1 domains and identified a hinge region that could be essential to this movement. Residues F12 and K47 seem to play a role in the intrinsic dynamics of TIMP-1. Interestingly, these residues are highly conserved in numerous mammal species (mouse, rabbit, horse…). This suggests the importance of these residues in TIMP-1 functions. *In silico* mutants have been proposed on these residues located in the hinge region, and their biological activity has been tested in mouse cortical neurons. We show that F12A or K47A mutations do not prevent TIMP-1 mutants from binding to LRP-1. Nevertheless, these mutations block their endocytosis and ability to reduce neurite length. Taken together, these results suggest that TIMP-1 binding to LRP-1 is followed by a TIMP-1 conformational change, which is required for its endocytosis and cellular effects.

In the absence of structural data regarding one of the two partners, considering the intrinsic dynamics of the proteins could provide additional information for studying ligand/receptor interactions and their associated cellular effects.

## Methods

### Molecular Modelling

NMA of the TIMP-1 protein was performed using the PDB 1UEA crystallographic structure. After the addition of hydrogen atoms, the structure was successively minimised using a combination of the Steepest Descent (1000 steps) and ABNR (50000 steps) methods. NMA was carried out using the Bio3D package within the Rstudio environment^27^. We used the calphax force field to account for stronger beta bridges and helix 1–4 interactions, specifying the six disulfide bridges (1–70, 3–99, 132–137, 145–166, 127–174, 13–124) when building the force constants. Graphs were generated with Rstudio.

A 100-ns-long molecular dynamics simulation in explicit solvent (TIP3P) was performed on the TIMP-1 minimised structure used for NMA with the NAMD software, along with the CHARMM27 force field, in the NPT ensemble at 293 K - 1 atm, with a cut-off of 11 Å for long-range interactions. Trajectory analysis and graphs were produced using the Bio3D package within the Rstudio environment^28^. Pictures were generated with PyMol software.

### Plasmid construction, production and purification of recombinant proteins

The pCMV6-XL5 vector containing TIMP-1 cDNA was obtained from Origene (Origene, Rockville, USA). Mutated TIMP-1 forms were generated at the F12 and K47 positions using the QuickChange II Site-directed Mutagenesis kit (Stratagene, Agilent Technologies, Les Ulis, France) with the following primers: F12A (forward): 5′-CCACCACAGACGGCCGCCTGCAATTCCGACCTCG-3′; (reverse): 5′-CGAGGTCGGAATTGCAGGCGGCCGTCTGTGGGTGG-3′, K47A (forward): 5′-GATGACCAAGATGTATGCAGGGTTCCAAGCCTTAGGG-3′; (reverse):5′-CCCTAAGGCTTGGAACCCTGCATACATCTTGGTCATC-3′. TIMP-1 and mutated cDNA were cloned into the p3XFLAG-CMV14 vector (Sigma-Aldrich, Saint Quentin Fallavier, France) to produce TIMP-1 with fusion FLAG-tag in C-terminal using the following primers: 5′- TACAGAATTCCACCATGGCCCC-3′ (forward) and 5′-GAGAGCTAGCGGCTATCTGGGACC-3′ (reverse). All constructs were confirmed by DNA sequencing analysis (Beckman Coulter Genomics, France). CHO cells were stably transfected with these constructs using Lipofectamine 2000 (Invitrogen, Cergy Pontoise, France) to produce FLAG-tagged TIMP-1 (T1-WT), FLAG-tagged TIMP-1 F12A (T1-F12A) and FLAG-tagged TIMP-1 K47A (T1-K47A). Cells were grown in DMEM-Ham’s F-12 medium with 10% (v/v) foetal bovine serum and 500 ng/ml geneticin at 37 °C in a humidified chamber containing 5% (v/v) CO_2_. The conditioned media were collected and incubated for 1 h with 10 mM EDTA in acidic conditions (pH 3.5) to remove the MMPs complexed to wild-type or mutated TIMP-1. After equilibration to pH 7.4 with NaOH, the conditioned media were incubated at 4 °C for 3 h with anti-FLAG M2 affinity gel (Sigma-Aldrich). The resin was then collected and washed 5 times with Tris-buffered saline (TBS, 50 mM Tris-HCl pH 7.4 containing 150 mM NaCl). The recombinant proteins were eluted under acidic conditions (0.1 M glycine-HCl pH 3.5), equilibrated at pH 7.4 by 1 M Tris pH 8.0 and dialysed against PBS. TIMP-1 wild-type and mutant concentrations were determined with the Human TIMP-1 Quantikine ELISA kit (R&D Systems, Lille, France). Recombinant protein purity and quantification were controlled by SDS-PAGE followed by silver nitrate staining and immunoblotting. Recombinant RAP was prepared as previously described^6^.

### Western blotting

Conditioned media and whole-cell protein extracts were prepared and analysed by western blotting using anti-TIMP-1 (1/500, Merck Biosciences), anti-FLAG (1/1000, mouse, clone M2, Sigma-Aldrich) or anti-actin (1/1000, Santa-Cruz Biotechnology), as previously described^6^.

### MMP inhibition assays

The inhibitory activities of T1-WT, T1-F12A and T1-K47A against MMP-1, -2, -3 and -9 were assayed as follows. All MMPs and substrates were obtained from Merck Biosciences.

*MMP*-*1*: proMMP-1 was activated by incubation in activation buffer (25 mM Hepes, 5 mM CaCl_2_, 20% (v/v) glycerol, 0.01% (v/v) Brij 35 and 4.5 mM APMA) for 2 h at 37 °C. More than 80% of the proMMP-1 was activated in these conditions (data not shown). The activated MMP-1 (2 nM) was then incubated for 1 h at 27 °C in Tris-test buffer (50 mM Tris-HCl pH 7.5 containing 150 mM NaCl and 5 mM CaCl_2_) containing 0.92 to 6.44 nM TIMP-1 or its mutated forms. The assay was initiated by adding MMP-1/-9 fluorogenic substrate DNP-Pro-Cha-Gly-Cys(Me)-His-Ala-Lys(N-Me-Abz)-NH_2_ (λ_exc_ = 365 nm/λ_em_ = 450 nm) at a final concentration of 5 µM.

*MMP*-*2*: 0.76 nM MMP-2 was incubated in Tris-test buffer containing 0.17 to 13.5 nM TIMP-1 or its mutated forms for 2 h at 27 °C before adding MMP-2/-7 fluorogenic substrate MCA-Pro-Leu-Gly-Leu-Dpa-Ala-Arg-NH TFA (λ_exc_ = 325 nm/λ_em_ = 393 nm) at a final concentration of 2.5 µM.

*MMP*-*3*: 2.3 nM MMP-3 catalytic domain was incubated with 0.1 to 5.4 nM TIMP-1 or its mutated forms in Tris-test buffer for 1 h at 27 °C. MMP-3 fluorogenic substrate NBD-Arg-Pro-Lys-Pro-Leu-Ala-Nva-Trp-Lys (DMC)-NH_2_ (λ_exc_ = 350 nm/λ_em_ = 465 nm) was added at a final concentration of 3 µM.

*MMP*-*9*: 0.3 nM MMP-9 was incubated in Tris-test buffer containing 0.7 to 11 nM TIMP-1 or its mutated forms for 2 h at 27 °C. The assay was initiated by adding MMP-1/-9 fluorogenic substrate (DNP-Pro-Cha-Gly-Cys(Me)-His-Ala-Lys(N-Me-Abz)-NH_2_ (λ_exc_ = 365 nm/λ_em_ = 450 nm) at a final concentration of 5 µM.

The rate of each substrate cleavage was measured in triplicate for each concentration examined, using an Infinite F200 PRO spectrofluorimeter (Tecan, Lyon, France) with one measure per minute for 20 min. Non-linear regression analysis with Graphpad software (La Jolla, USA) allowed us to calculate the K_i_ using the Morrison equation^43^.

### Surface plasmon resonance analysis

Surface plasmon resonance experiments were performed using Biacore T200 (GE Healthcare Life Sciences, Velizy-Villacoublay, France) as previously described^6^. Injections were performed with HBS-N buffer (50 mM Hepes, 150 mM NaCl, Tween 0.005% (v/v), pH 7.4). Recombinant LRP-1 mini-domains DII and DIV (R&D) with an Fc-tag were immobilised on a CMD500m sensor chip (Xantech) functionalised with anti-Fc antibody. Different concentrations of TIMP-1 and of its mutated forms (5–160 nM) were injected in multi-cycle kinetics to allow determination of the association (*k*_*on*_) and dissociation (*k*_*off*_) constants. EGF was used as the negative control.

### Neuron morphological analysis

Primary cultures of cortical neurons were prepared from CD1 mice embryos as previously described^6^ and cultured as previously described^30^. These experiments involving mice were carried out in accordance with the French and European guidelines for the care and use of laboratory animals and were approved by the ethic committee of the University of Reims Champagne-Ardenne (Protocol number: 1A08248094720). According to the specific biological activity of TIMP-1 in neurons^6, 30^, we chose to use a concentration of 5 nM of TIMP-1, which allowed TIMP-1 quantification in the endocytosis assay. Five nM of T1-WT, T1-F12A or T1-K47A or mixtures of T1-WT and mutants with different ratios were added to the cultures in serum-free media for 30 min. The neurons were rinsed with PBS and fixed in 4% (v/v) paraformaldehyde for 10 min. Immunofluorescence studies with anti-βIII-tubulin antibodies (1/100, clone SDL.3D10, Sigma-Aldrich) were performed as previously described^30^. The cells were observed using an Olympus BH2-RFCA fluorescent microscope with a 40x objective lens and photographed with a U-MPTV Camera using the DP Controller software (all from Olympus). In each experiment, fluorescence microphotographs were taken from at least 5 randomly selected fields per slide, with 4 slides per experimental condition (total 20 fields). The neurite length corresponded to the average length of the neurites of each cell and was obtained by dividing the total length of the neurites in a field by the number of neurite segments, as previously described^6^. The neurite length of all βIII-tubulin positive cells was measured using the ImageJ plugin NeuronJ. A neurite segment was defined as the distance between branching points or the distance between the branching point and the top of a neurite.

### Fluorescent TIMP-1 preparation and endocytosis experiment

Ten µg of T1-WT, T1-F12A or T1-K47A were biotinylated with the ProtOn Biotin Labelling Kit according to the manufacturer’s instructions. The biotinylated products were incubated with 0.5 µg eFluor® 450 Streptavidin (eBioscience) for 30 min at RT. The neuron-surface binding and endocytosis of 5 nM fluoT1-WT, fluoT1-F12A or fluoT1-K47A were studied as previously described^6^. Briefly, cortical neurons plated in 24-well plates were incubated in assay medium (culture medium containing 0.1% (v/v) bovine serum albumin, BSA) at 4 °C for 1 h. For the binding assays, cells were incubated with 5 nM fluo-T1 (-WT, -F12A and -K47A) at 4 °C for 2 h in the absence or presence of 500 nM RAP or 30 µg/mL blocking LRP-1 polyclonal antibodies (R2629)^34^. The cells were then carefully rinsed five times with cold PBS and surface-digested with 0.1% (w/v) pronase in culture medium at 4 °C to degrade the surface-bound ligands and to detach cells. After cell collection by centrifugation, fluorescence in the supernatant corresponding to the surface-bound ligand and fluorescence in the pelleted cells corresponding to the internalised ligand were measured by spectrofluorimetry (λexc: 405 nm/λem: 445 nm) (Infinite F200 PRO, Tecan, Lyon, France). Appropriate controls using only eFluor were performed. For the endocytosis assays, after fluo-T1 binding (as described above), the cells were carefully rinsed with PBS and further cultured in assay medium pre-warmed at 37 °C for 15 min. To distinguish surface binding from intracellular accumulation, the cells were washed twice with cold PBS and surface-digested with pronase as described above.

### Confocal microscopy analysis of LRP-1 mediated endocytosis

Neurons were first equilibrated at 4 °C for 30 min with cold serum-free defined medium supplemented with 1 mg/ml BSA to saturate the cell surface. Five nM of T1-WT, T1-F12A or T1-K47A were then added for 60 min at 4 °C in the presence or absence of 500 nM RAP. Endocytosis was allowed by incubation at 37 °C for 10 min or 1 hour and stopped by replacing the cells at 4 °C. The neurons were extensively rinsed with cold PBS and fixed in 4% (v/v) paraformaldehyde for 10 min. For immunofluorescence, cells were incubated with anti-TIMP-1 (1/300, Merck Biosciences) and anti-EEA1 (1/300, Abcam) at 4 °C overnight, followed by secondary antibodies conjugated to Alexa Fluor 488 (1/1000, green, Molecular Probes) or to Alexa Fluor 568 (1/1000, red, Molecular Probes) at room temperature for 60 min. After rinsing with PBS, the slides were mounted in fluorescence mounting medium. Acquisitions were made with a confocal microscope LSM710 with a 63x objective and the Zeiss operating system associated with the ZEN software program (all from Carl Zeiss MicroImaging GmbH, Germany). Acquisitions were performed by exciting Alexa Fluor 488 and Alexa Fluor 568 with an argon laser, a helium-neon laser and a chameleon infrared laser tuned at 730 nm. Emitted fluorescence was detected through the appropriate wavelength window. In each experiment, fluorescence microphotographs were taken from at least 10 randomly selected fields per slide, with 3 slides per experimental condition (total 30 fields). In each field, 20 images were captured with a 0.3 μm z-step. Maximal intensity projection representations were realised using the AMIRA software program (v5.4.2, Visualization Sciences Group, Burlington, MA, USA). For the colocalisation studies, a multichannel field module was used, followed by a correlation plot treatment (subrange values of 15 to 255; gamma, 0.5). Then, an isosurface representation of the correlation plot was realised using the same threshold for each treatment. The TIMP-1 average intensity of fluorescence per µm^2^ for each cell was performed using the particle analysis function of the ImageJ software. Quantification of the TIMP-1/EEA1 colocalisation was performed using the AMIRA software program.

### Statistical analysis

Statistical analysis was performed using a Student’s t-test or one-way ANOVA with a Newman Keuls post-test (GraphPad Prism version 6.01 for Windows, GraphPad Software, San Diego California USA, www.graphpad.com). The values represent the mean ± SD of at least three independent experiments. P-values of <0.05, <0.01, <0.001 and <0.0001 are identified with *, **, *** and ****, respectively. NS represents a value that is not statistically significant.

## Electronic supplementary material

Supplementary information
