# Supplementary material for: Intrinsic dynamics study identifies two amino acids of TIMP-1 critical for its LRP-1-mediated endocytosis in neurons
Source: Sci Rep. 2017 Jul 14;7:5375. doi: 10.1038/s41598-017-05039-z (PMC5511134; doi:10.1038/s41598-017-05039-z)
Supplement: Supplementary file 1 — Supplementary information [file 41598_2017_5039_MOESM1_ESM.pdf]

Intrinsic dynamics study identifies two amino acids of TIMP-1 critical for its LRP-1-mediated endocytosis in neurons

Laurie Verzeaux[1], Nicolas Belloy[1,2], Jessica Thevenard-Devy[1], Jérôme Devy[1], Géraldine Ferracci[3], Laurent Martiny[1], Stéphane Dedieu[1], Manuel Dauchez[1,2], Hervé Emonard[1], Nicolas Etique[1,§,*] & Emmanuelle Devarenne-Charpentier[1,§,*]

[1]CNRS UMR 7369: Matrice Extracellulaire et Dynamique Cellulaire (MEDyC), UFR Sciences Exactes et Naturelles, Université de Reims Champagne-Ardenne (URCA), Laboratoire SiRMa - Campus Moulin de la Housse, BP 1039, 51687 Reims cedex, France

[2]Plate-forme de Modélisation Moléculaire Multi-échelle (P3M), Université de Reims Champagne-Ardenne, Reims, France

[3]Aix-Marseille Université, CNRS, Centre de Recherche en Neurobiologie et Neurophysiologie de Marseille (CRN2M), UMR 7286, Plate-forme de Recherche en Neurosciences (PFRN), Marseille, France

[§]These authors contributed equally to this work

*Corresponding author: nicolas.etique@univ-reims.fr

SUPPLEMENTARY VIDEO LEGENDS

Supplementary Movie 1. TIMP-1 intrinsic dynamics along the modes 7, 8 and 9. The displacements of the TIMP-1 backbone along the first three modes (Mode 7, Mode 8 and Mode 9) calculated by NMA are visualized.

SUPPLEMENTARY FIGURES

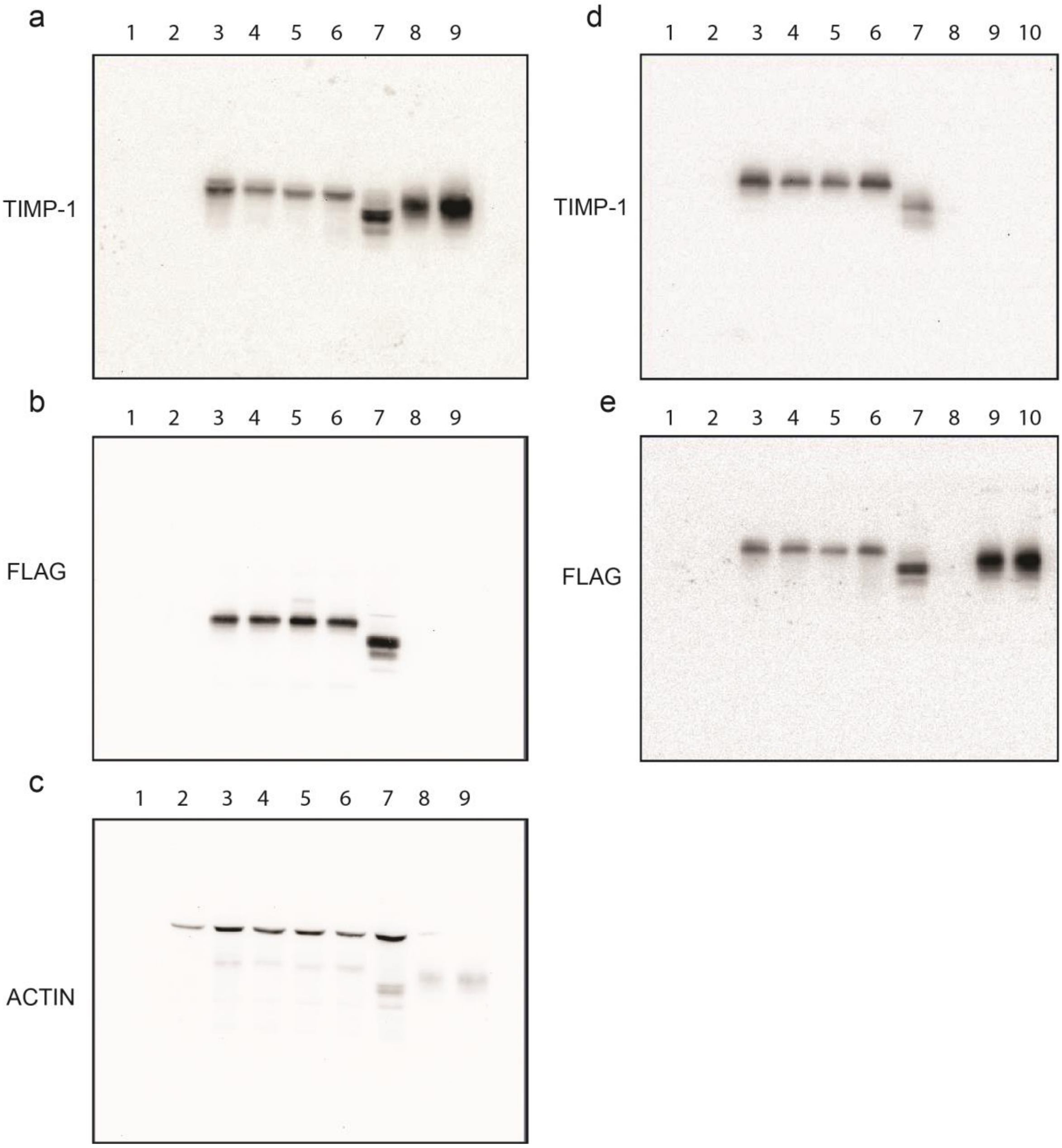

Figure S1: Complete blots with the indicated antibodies presented in the figure 4 of the manuscript. Panel **a**, **b** and **c** correspond to blots of cell-extracts and panel **d** and **e** correspond to blots of conditioned medium. For the panel **a**, **b** and **c**: lane 1- PageRuler Prestained Protein Ladder; 2- cell extract of non-transfected CHO cells; 3- cell extract of CHO cells transfected with p3X-FLAG-CMV-14 expressing T1-T2G (mutant unpresented in this manuscript); 4- cell extract of CHO cells transfected with p3X-FLAG-CMV-14 expressing T1-WT; 5- cell extract of CHO cells transfected with p3X-FLAG-CMV-14 expressing T1-F12A; 6- cell extract of CHO cells transfected with p3X-FLAG-CMV-14 expressing T1-K47A; 7- cell extract of CHO cells transfected with p3X-FLAG-CMV-14 expressing N-TIMP-1 (N-terminal domain of TIMP-1: AA 1 to 125); 8- 10 ng of Recombinant TIMP-1 (Merck Millipore); 9- 50

ng of Recombinant TIMP-1. For the panel **d** and **e**: lane 1- PageRuler Prestained Protein Ladder; 2- Conditioned medium of non-transfected CHO cells; 3- Conditioned medium of CHO cells transfected with p3X-FLAG-CMV-14 expressing T1-T2G (mutant unpresented in this manuscript); 4- Conditioned medium of CHO cells transfected with p3X-FLAG-CMV-14 expressing T1-WT; 5- Conditioned medium of CHO cells transfected with p3X-FLAG-CMV-14 expressing T1-F12A; 6- Conditioned medium of CHO cells transfected with p3X-FLAG-CMV-14 expressing T1-K47A; 7- Conditioned medium of CHO cells transfected with p3X-FLAG-CMV-14 expressing N-TIMP-1 (N-terminal domain of TIMP-1: AA 1 to 125); 9- 10 ng of Recombinant TIMP-1; 10- 50 ng of Recombinant TIMP-1
